# A Full-Body Motion Capture Gait Dataset of Healthy Young Adults Walking Ramps Up and Down

**DOI:** 10.1038/s41597-025-06535-y

**Published:** 2026-01-08

**Authors:** Johanna Vielemeyer, Lisa Tronicke, Lucas Schreff, Rainer Abel, Knut Lechler, Roy Müller

**Affiliations:** 1 Department of Orthopedic Surgery, Klinikum Bayreuth GmbH, Bayreuth, Germany; 2 Institute of Sport Sciences, Friedrich Schiller University Jena, Jena, Germany; 3 Medical Office, R&D, Össur ehf, Iceland; 4 Universitätsklinikum Erlangen, Friedrich-Alexander-Universität, Erlangen, Germany; 5 Bayreuth Center of Sport Science, University of Bayreuth, Bayreuth, Germany

**Keywords:** Bone quality and biomechanics, Biomedical engineering

## Abstract

3D motion capture data of healthy participants is of high value for research groups working in basic research, clinical settings, or the development of medical devices as a reference or for comparison purposes. This dataset provides kinematic and kinetic data from 13 healthy young adults (five women, eight men; mean age 28.2 ± 4.7 years) during ramp walking at three inclinations (0°, 7.5°, and 10°). Using a 3D motion capture system (Vicon Motion Systems Ltd) consisting of 10 infrared cameras, two video cameras and three force plates (Advanced Mechanical Technology, Inc.), data was recorded as participants walked up and down a 6 m instrumented ramp at self-selected speeds. In each trial, two consecutive strides over the force plates are captured. The dataset includes raw data and processes kinematic and kinetic data of the lower limb and upper body based on the Plug-in Gait full body model.

## Background & Summary

With the advancement of technology, the use of 3D motion capture has become a state-of-the-art methodology to measure and visualize human biomechanics. It is fundamental to the analysis and understanding of the gait strategies of both able-bodied individuals and those with pathological movement patterns which may be caused by various reasons e.g. diseases, injuries, or medical interventions^1–5^. A structured overview of existing biomechanical datasets from several fields of research can be found on Github (https://github.com/mkjung99/biomechanics_dataset).

When researching pathological movement patterns, one common approach is comparing physiological movement patterns to identify differences. Access to datasets of healthy, able-bodied individuals is an important contribution, supporting research teams in their work. For the development of medical devices designed to assist users on various terrains such as level ground and inclines/declines, biomechanical data for those terrains including both kinematic and kinetic data are relevant to optimize product design. These data are also important to achieve improved user experience and more physiological gait. In the field of exo-prosthetic devices, decline angles of 7.5° and higher are of particular interest as they place not only special demands on the performance and function of the devices^6,7^ but also on the user^8^.

**Fig. 1:**
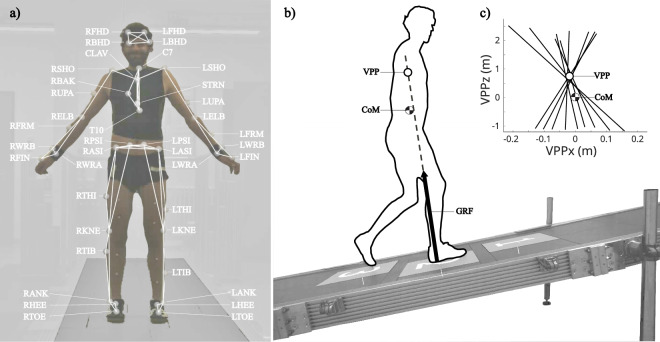
(**a**) Plug-in Gait full body marker set. (**b**) The subject walks up a ramp with three embedded force plates (marked with 1, 2 and 3), measuring ground reaction forces (GRFs). During the single support phase of human walking, GRFs in the sagittal plane are typically aligned toward a virtual point situated above the body center of mass (CoM)^17^. This point is known as the virtual pivot point (VPP) and reflects a postural control strategy whereby the resultant GRF vector is aligned to stabilize the upper body. (**c**) Example VPP plot in a CoM-centered coordinate frame. The position of the VPP (horizontal: VPPx, vertical: VPPz) is calculated using GRF vectors starting at the center of pressure for every instant measurement. The VPP position is defined as the point where the sum of the squared perpendicular distances to the GRFs is minimal. Vielemeyer *et al*.^18^ provides a detailed description of VPP calculation.

Existing data sets include kinetic and kinematic data of human ramp walking^9–12^ with variations in the number of force plates, measured joints, biomechanical variables, or additional activities performed. Most studies report joint data of the lower body^9,10,12^. However, dynamic stability of the whole body during walking is strongly dependent on the upper body movements^13^. Most upper body gait parameters are affected by the ramp angle^10^, highlighting the critical role of upper body dynamics in inclined ambulation. Only the full-body kinematics provided by underlying dataset enable calculation of biomechanical parameters such as whole body angular momentum^14^, the margin of stability^15,16^, the virtual pivot point^17,18^ (VPP, Figs. 1c, 6) and the collision fraction^19,20^, all based on the center of mass. Collectively, these parameters provide comprehensive insights into balance control, dynamic stability, and mechanical energy dissipation. Our dataset was recorded during free walking over two consecutive force plates whereas others used only one force plate^10,11^ or instrumented treadmills^12,21^. Differences in data collection methods, especially between free walking and treadmill use, can impact results^22,23^, which may be relevant for clinical studies.

This paper presents a comprehensive full-body dataset of 3D motion capture data, including marker data, force plate data of two consecutive contacts, gait events, and anthropometric data of 13 healthy young adults walking up and down a ramp (0°, 7.5°, and 10°) at self-selected walking speeds. An established marker set (Plug-in Gait full body) was used, ensuring that the data can be processed by gait lab teams that work with a Vicon system and the associated software. This improves accessibility, enabling gait lab teams to process data without requiring advanced programming skills or resources. The dataset is versatile and can be used to develop or improve control algorithms for prostheses, exoskeletons, or humanoid robots, as well as for basic research on human walking. Additionally, it may serve as a reference data set for clinical studies, extending the pool of publicly available data sets.

## Methods

### Participants

This study was conducted on 13 healthy young adult participants (five women, eight men) with a mean age of 28.2 ± 4.7 years, mean body mass of 73.3 ± 12.4 kg and mean height of 1.78 ± 0.09 m without any known impairments that could impact their gait pattern (Table 1).

**Table 1:** Anthropometric data of participants.

		Ref01	Ref02	Ref03	Ref04	Ref05	Ref06	Ref07	Ref08	Ref09	Ref10	Ref11	Ref12	Ref13
age [y]		28	34	24	26	24	27	37	26	25	38	25	24	29
gender		m	m	f	m	f	m	m	m	f	f	m	m	f
weight [kg]		65	93	62	70	67	67	90	67	57	68	65	96	85
height [mm]		1760	1790	1730	1840	1690	1800	1850	1830	1710	1580	1830	1940	1680
ASIS distance [mm]		235	263	246	210	204	226	264	226	223	265	222	299	255
leg length [mm]	left	945	950	890	944	877	973	945	985	884	847	1010	1015	885
	right	950	965	890	942	875	969	942	985	877	845	1013	1010	880
knee width [mm]	left	94	126	109	99	111	103	120	100	112	117	95	126	125
	right	97	126	110	99	113	100	120	101	112	113	99	124	120
ankle width [mm]	left	68	74	67	70	69	75	76	71	68	59	70	74	68
	right	72	68	68	62	66	76	77	67	66	61	68	72	66
shoulder offset [mm]	left	45	52	45	45	40	45	50	45	45	40	45	60	45
	right	45	52	45	45	40	45	50	45	45	40	45	60	45
elbow width [mm]	left	78	96	65	90	80	82	88	88	78	77	81	95	92
	right	78	96	65	90	80	82	88	88	78	77	81	95	92
wrist width [mm]	left	54	62	57	67	58	62	61	62	58	59	59	68	58
	right	54	62	57	67	58	62	61	62	58	59	59	68	58
hand thickness [mm]	left	28	31	25	30	30	32	30	28	27	25	28	34	28
	right	28	31	25	30	30	32	30	28	27	25	28	34	28

### Experimental protocol

Prior to participation, all participants were informed about the study procedures. By signing the informed consent form, they agreed to participate and permitted the use of the generated data for publication. The protocol was approved by the ethics committee of the University of Jena (5368-12/17) and was conducted in accordance with the Declaration of Helsinki.

Participants walked up and down a 6 m instrumented ramp at inclinations of 0°, 7.5°, and 10° at self-selected speed. Three force plates, positioned in the middle section of the ramp, recorded ground reaction forces. The ramp was stabilized with support to minimize vibrations. A 1 m level platform was located at the end of the ramp.

### Instrumentation and participant preparation

Biomechanical data were collected using a 3D gait system from Vicon Motion Systems Ltd, consisting of ten infrared cameras (Vero v2.2) and two video cameras (Vue) with a sampling rate of 200 Hz, and three force plates (AMTI Optima BMS464508) with a sampling rate of 1000 Hz. The camera system and force plates were synced.

All participants wore shoes (Adidas Samba) provided by the lab for all measurements. Preparation for the 3D gait analysis followed the standard procedure for the Plug-in Gait Full body marker set, including marker placement and taking subject measurements (Fig. 1a, Table 2). Foot markers except for the ankle marker were placed on the shoe.

**Table 2:** Marker labels and positions of the full body Plug-in Gait.

	Marker Label	Definition	Position
Head	LFHD	Left front head	Left temple
	RFHD	Right front head	Right temple
	LBHD	Left back head	Left back of head
	RBHD	Right back head	Right back head
Thorax	C7	Seventh cervical vertebrae	Base of the neck
	T10	Tenth thoracic vertebrae	Centre mid-back
	CLAV	Clavicle	Top of the breastbone
	STRN	Sternum	Base of the breastbone
	RBAK	Right back	Centre of the right shoulder blade
Upper limb	LSHO/RSHO	Left/right shoulder	Acromion-clavicular joint
	LUPA/RUPA	Left/right upper arm	Laterally on upper arm
	LELB/RELB	Left/right elbow	Lateral epicondyle
	LFRM/RFRM	Left/right forearm	Laterally on forearm
	LWRA/RWRA	Left/right wrist	Hand wrist on the thumb side using a wrist bar
	LWRB/RWRB	Left/right wrist	Hand wrist little finger side using a wrist bar
	LFIN/RFIN	Left/right finger	Proximal to the middle knuckle on the hand
Pelvis	LASI/RASI	Left/right anterior superior ilia	Bony prominence on the frontal pelvis
	LPSI/RPSI	Left/right posterior superior ilia	Bony prominence on the lower back
Lower limb	LKNE/RKNE	Left/right knee	lateral distal epicondyle of the femur
	LANK/RANK	Left/right ankle	Prominence of the lateral malleolus
	LTHI/RTHI	Left thigh Lateral	Between KNE and trochanter
	LTIB/RTIB	Left tibia lateral	Between KNE and ANK
	LTOE/RTOE	2^nd^ Toe Proximal	forefoot over second metatarsal head
	LHEE/RHEE	Left heel	Calcaneus (same height as TOE)

### Experimental procedures and data collection

After preparing the participants for the 3D gait analysis, they were asked to walk up and down the ramp several times to familiarize themselves with the inclination (Fig. 1b). The order of the different inclinations was randomized for each participant. Participants were not informed about the force plates’ location to avoid targeting; however, the tester observed the participants’ strides and recommended a starting point to increase the probability of valid force plate contacts.

For each condition, a static trial while standing still was recorded and labeled before dynamic measurements. Participants walked at self-selected walking speed. Incline and decline trials were recorded alternately. Subjects reached the force plates after approximately three steps. Twelve valid trials were recorded for each condition. A trial was considered valid when the left and right foot each hit a force plate without overstepping.

### Data processing

The raw data was processed in Nexus 2.16.0 × 64 (Vicon Motion Systems Ltd). For labelled trajectories, gap filling and filtering (mean square error setting) based on Woltring^24^ was used. Gait events were initially identified based on the detected events (heel strike and toe off) on the force plate with a force threshold of 20 N. Furthermore, the ankle and toe marker position was used for event detection and assigning right or left side run by the software. Values below 20 N would be too far below the noise floor level of the system setup (25 N) for adult participants^25^, greater values would reduce accuracy^26^. For all gait events, it was double checked if the force plate event detection and the second heel strike in a stride matched the event shown on video. For calculation of all biomechanical parameters, the Plug-in Gait full body model was run. Model data was then exported as c3d and txt. A list of all output variables can be found on Github (file *Variables.pdf*, https://github.com/jvielemeyer/human-ramp-walking).

## Data Records

**Fig. 2:**
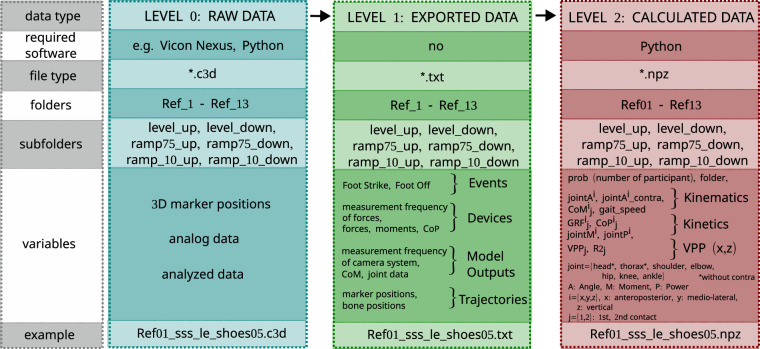
Data processing workflow diagram. Raw data was processed with Vicon Nexus and Python software to result in calculated and compressed (*.npz) data.

All data files are published in FigShare^27^. The repository contains three folders, corresponding to three levels of data: (1) Raw data in c3d format, (2) exported raw data in txt format, (3) calculated data, including biomechanical variables packed in npz format (For illustration see Fig. 2). All three main folders contain subfolders named Ref_01, Ref_02, etc. Then again, these subfolders are separated into folders named after the six experimental setups: level_up, level_down, ramp_75_up, ramp_75_down, ramp_10_up, ramp_10_down (see Fig. 2). Here, “ramp_75” denotes walking over a ramp of 7.5°, and “ramp_10” denotes walking over a ramp of 10°.

### Level 0: Raw data

Raw kinematic and kinetic data were stored in c3d format, which can be opened with Nexus (software of Vicon), MlsViewer or Mokka, as well as with processing software such as Visual3D.

For each trial, there is one file. Data of two participants were excluded because of incomplete data sets (see section of Missing data. For Ref. ^2^, ramp 10° up there exists only ten instead of twelve trials. That means, there are altogether 11 × 5 × 12 - 2 = 658 files (participant x setup x number of trials per setup – missing trials), since level_up and level_down contain only 6 trials each (because of the equality of these two conditions; note that for level walking “up” and “down” only term the walking direction). For example, the c3d file for participant 1, level_up, first trial is called *Ref01_sss_le_shoes04.c3d*.

### Level 1: Exported data

Raw kinematic and kinetic data were exported to txt files. They are stored in the folder Data_Level_1 and are arranged in the same structure as the Level 0 data. The files are called e.g. *Ref01_sss_le_shoes04.txt*. A data file of level 1 contains four parts: (1) “Events”, which includes time points of foot strike ( = heel strike) and foot off ( = toe off), (2) “Devices” with information about the raw kinetic data (measurement frequency of force plates, forces, moments, center of pressure (CoP)), (3) “Model outputs” with variables that were calculated by the system (e.g. measurement frequency camera system, center of mass (CoM), joint angle (A)/ force/ moment (M)/ power (P)), (4) “Trajectories” with raw kinematic data (measurement frequency camera, marker positions, bone positions).

### Level 2: Calculated data

Chosen computed biomechanical variables were packed in npz format. They are stored in the folder Data_Level_2 and are arranged in the same structure as raw data. The files are called e.g. *Ref01_sss_le_shoes04.npz* and can be opened with python.

The variables are (1) kinematic variables: angle of head, thorax, shoulder, elbow, hip, knee and ankle joint, and CoM (all normalized to gait cycle); gait speed, (2) kinetic variables: GRFs and CoP of two contacts, normalized to contact time, joint moment, and joint power, (3) VPP and R² in sagittal plane for two contacts. Only the last-mentioned variables (VPP and R²; using calculation instructions of Maus *et al*.^17^, Gruben and Boehm^28^, and Herr and Popovic^14^) and the gait speed were calculated manually; all other biomechanical variables were taken from the model (Nexus output).

## Technical Validation

### Calibration

Prior to each session, the cameras were calibrated according to the calibration process defined by the system’s manufacturer. After each change of the ramp inclination, the location of each force plate was determined and saved to the system by using the calibration wand which was placed at a corner of each force plate.

### Visualization

**Fig. 3:**
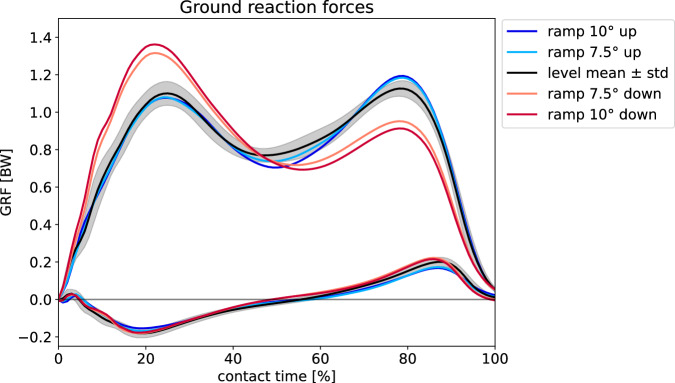
Ground reaction forces (GRFs) normalized to body weight (BW) for level (black) and ramp (10° up (blue), 7.5° up (light blue), 7.5° down (light red), and 10° down (red)) walking. The curves represent the mean over all trials and all participants for each condition; for level walking, also the standard deviation is shown (gray shaded area). The contact time is the normalized time from heel strike to toe off of the same leg. The lower (higher) curves represent the anteroposterior (vertical) GRFs.

**Fig. 4:**
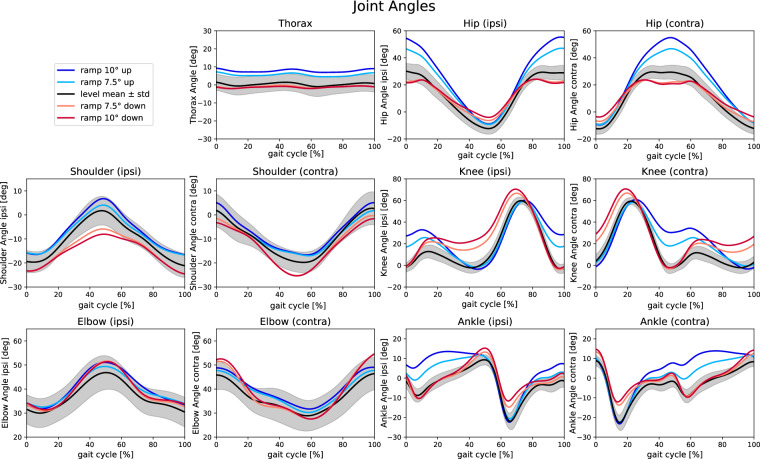
Joint angles for level (black) and ramp (10° up (blue), 7.5° up (light blue), 7.5° down (light red), and 10° down (red)) walking. The curves represent the mean over all trials and all participants for each condition; for level walking, also the standard deviation is shown (gray shaded area). One gait cycle is the normalized time between two consecutive heel strikes of the same leg. The ipsilateral (contralateral) limbs are denoted by “ipsi” (“contra”). Positive (negative) values indicate flexion (extension).

**Fig. 5:**
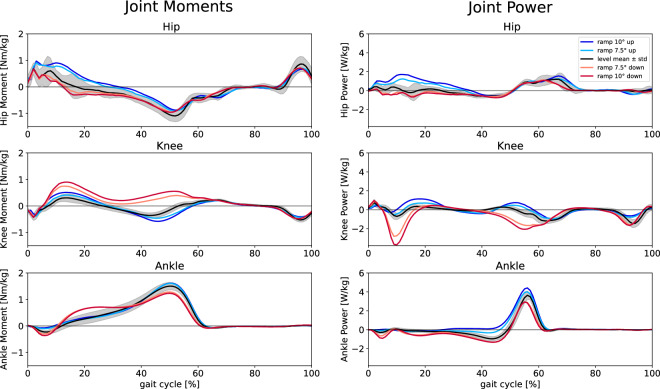
Joint moments (left side) and power (right side) of the lower body (ipsilateral leg) for level (black) and ramp (10° up (blue), 7.5° up (light blue), 7.5° down (light red), and 10° down (red)) walking. The curves represent the mean over all trials and all participants for each condition; for level walking, also the standard deviation is shown (gray shaded area). One gait cycle is the normalized time between two consecutive heel strikes of the same leg. For moments, positive (negative) values indicate flexion (extension).

**Fig. 6:**
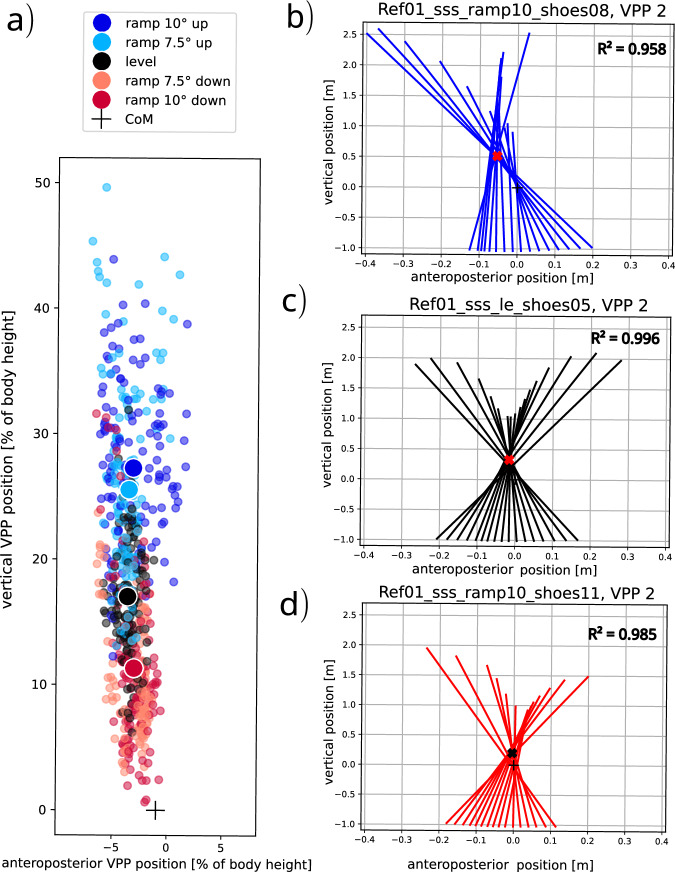
(**a**) Each small dot represents the virtual pivot point (VPP) position of the single support phase of one trial (the second one of the two consecutive contacts) in a center of mass centered, vertically aligned coordinate frame (horizontal: anteroposterior VPP position relative to body height, vertical: vertical VPP position relative to body height). The positions are normalized to body height of the participant. All trials of all participants are shown. The bigger dots show the mean values over all trials and participants (ramp 10° up (blue), ramp 7.5° up (light blue), level (black), ramp 7.5° down (light red), and ramp 10° down (red)). (**b**) - d) Exemplarily VPP plot of one participant (for construction details see explanation of Fig. 1c, horizontal: anteroposterior VPP position, vertical: vertical VPP position) for ramp 10° up (**b**), level (**c**), and ramp 10° down (**d**).

We analyzed, visualized, and validated data in Python (see Code Availability). The python script *main_visualization.py* may be used to create i.a. (1) a plot of horizontal and vertical GRFs (Fig. 3), (2) a 3 × 4 plot of the angle of thorax, shoulder, elbow, hip, knee and ankle (ipsi- and contralateral, Fig. 4), and (3) a 3 × 2 plot of the moment and power of hip, knee and ankle (Fig. 5). Mean values for all trials and participants are shown. To create plots of single trials, the VPP calculation tool GUI can be used (as illustrated for VPP in Fig. 6b-d, see Usage Notes).

### Missing data

Two participants, Refs. ^3,4^, did not manage to hit a force plate with left and right foot for all trials, each without overstepping. As kinetic data are missing for almost all setups, these participants were not included in the analyses. However, the raw data of the correct trials of Refs. ^3,4^ can be found on FigShare^27^ in a separate folder.

### Comparison to other data

We did not find a single data set that presents the variables of the full body model for ramp walking. Thus, we compared the data with several studies separately. To assess the validity of lower body joint data, we considered the presented plots of Carmargo *et al*.^10^ (overground walking, 1.17 ±0.21 m/s, ±7.8° and ±9.2° ramp), Reznick *et al*.^12^ (treadmill, 1.0 m/s, ±10° ramp) and Dimitrov *et al*.^9^ (overground walking, self-selected speed, ±5° and ±15° ramp). The walking speeds of these data sets are comparable to our study (level: 1.3 ±0.1 m/s, ramp walking. 1.3 ±0.1 m/s) The curves were in shape and extreme values comparable to our curves. For instance, during ramp ascent, increasing inclination leads to increased hip and knee flexion, especially in early stance and late swing phases. It also leads to greater ankle dorsiflexion during the stance phase. During ramp descent, steeper inclination reduces hip extension, while knee flexion increases mainly during loading response and mid stance. Data for joint angles of the upper body (elbow, shoulder and thorax) at level walking (comfortable walking speed) were presented by Romkes *et al*.^29^ and are comparable to our data.

To validate the ground reaction forces, we found the study of Lay *et al*.^11^, including uphill and downhill walking of a ramp of 15° at self-selected speed. Here, the curve shapes are comparable to ours for the vertical component.

We observed consistency within and across participants (for visual validation use the section “plot single trials” in main_visualization.py). We calculated the virtual pivot point (VPP) as described in the explanation of Fig. 1c to validate the measured data. For level walking, the mean VPP is placed at −2.3% of body height (anteroposterior position) and 17% of body height (vertical position), as illustrated in Fig. 6a. This is comparable to the VPP values measured in former studies. The anteroposterior position lies in some studies at 0% of body height^18,30^, also values of –0.2%^31^, 0.1%^32^, and 0.2%^33^ of body height were reported. The vertical VPP is positioned at 11%^33^, 12%^30^, 14%^32^, and 16%^18,31^ of body height (if absolute values were given in the literature, they were normalized to mean body heights to enable comparability). The GRFs point very close to the calculated VPP (R² > 97% (mean value, for each condition separately) for all conditions, exemplarily illustrated in Fig. 6b-d). This is also an indication of the high validity of the data, and it is for level walking comparable with the above-mentioned literature (R² > 97% if calculated).

### Limitations

It should be mentioned that the number of participants (N = 13) and their variety in characteristics such as age and BMI are limited. The sample is skewed towards male participants (8 males, 5 females). For analyses requiring a balanced gender distribution, it is possible to include only five participants from each gender, as the repeated measurement design supports reliable analysis of biomechanical parameters.

The force plate setup required for kinetic data limits analysis to two consecutive steps. However, compared to datasets using a single force plate, this approach allows evaluation of kinetic parameters over two consecutive strides, which is valuable for analyzing the double support phase. Walking speed was not controlled between subjects and incline levels. Walking speed effects joint loading and spatio-temporal parameters during uphill and downhill^34^ walking.

## Usage Notes

The code is separated into a calculation and a visualization part. The structure is explained in the README file (https://github.com/jvielemeyer/human-ramp-walking). The files *main_calculation.py* and *main_visualization.py*, and the initialization files are stored in the main folder. The code *main_calculation.py* starts a calculation GUI. The usage of the GUI is explained in the file *Documentation_gui.pdf*. In the document *Variables.pdf*, all output variables of the Vicon system are listed.

The input data is stored in the folder Data_Level_1. Possible output of running *main_calculation.py* is: (1) compressed (*.npz) files of all calculated data (see section Data Records), (2) table (*.csv) files with VPP values of each trial, (3) plots of single trials for GRFs, CoP, CoM, VPP and joint variables.

The input of the visualization part is stored in the folder Data_Level_2. The output of *main_ visualization.py* includes (1) mean plots of kinematics and GRFs, (2) boxplot of VPP, (3) table (*.csv) file with mean and standard deviation values of VPP.

## Data Availability

The dataset is available on Figshare: 10.6084/m9.figshare.29300888.v1.

## Code availability

The code is available on GitHub: https://github.com/jvielemeyer/human-ramp-walking.
